# Positive correlation between gene coexpression and positional clustering in the zebrafish genome

**DOI:** 10.1186/1471-2164-10-42

**Published:** 2009-01-22

**Authors:** Yen Kaow Ng, Wei Wu, Louxin Zhang

**Affiliations:** 1 Department of Mathematics, National University of Singapore, 2 Science Drive 2, Singapore 117543, Singapore; 2 Institute of Molecular and Cell Biology, Singapore 138673, Singapore

## Abstract

**Background:**

Co-expressing genes tend to cluster in eukaryotic genomes. This paper analyzes correlation between the proximity of eukaryotic genes and their transcriptional expression pattern in the zebrafish (*Danio rerio*) genome using available microarray data and gene annotation.

**Results:**

The analyses show that neighbouring genes are significantly coexpressed in the zebrafish genome, and the coexpression level is influenced by the intergenic distance and transcription orientation. This fact is further supported by examining the coexpression level of genes within positional clusters in the neighbourhood model. There is a positive correlation between gene coexpression and positional clustering in the zebrafish genome.

**Conclusion:**

The study provides another piece of evidence for the hypothesis that coexpressed genes do cluster in the eukaryotic genomes.

## Background

In most eukaryotes, the transcription factor mechanism seems sufficient to ensure coregulation of genes, and hence co-localization of genes is not critical. Accordingly, there should be no selection pressure for coregulated genes to line up next to each other in an eukaryotic genome. However, genes are not randomly distributed in the genome as they were thought to be even after tandem genes are excluded [1,2]. The coexpression of clustered genes has been reported in *Homo sapiens *[3], *Caenorhabditis elegans *[4-7], *Drosophila melanogaster *[8-12], *Saccharomyces cerevisiae *[8,13,14], and *Arabidopsis thaliana *[15]. Moreover, positional clustering of genes that are highly expressed in a specific tissue or a pathway has also been revealed in different genomes [16-19].

These mentioned studies on the coexpression of proximate genes and positional clustering of coexpressed genes are based on expression data obtained from biotechnologies such as SAGE data, DNA microarray, together with gene annotations. There are several reasons for proximate genes to be coexpressed to a certain degree. There are operons in *C. elegans *[4,20]. Adjacent gene pairs can share *cis*-regulatory elements [21]. There could be some selection force that keeps coregulated genes in the same region, for example, to make transcription more efficient as a group [10].

Here we present a genome-wide analysis of clustering of coexpressed genes in the zebrafish genome using available microarray data. As a representative of the bony fishes, the zebrafish has become a well-established model organism in a variety of studies in developmental biology and drug discovery. It has made important contributions to the identification of genes involved in development, behaviour and disease. The zebrafish genome is about 1.9 billion base pairs long and contains approximately 20,000 to 30,000 genes on 25 chromosomes. We first used a method proposed in William and Bowles [15] to examine the degree of coexpression among proximity genes. We investigated the effect of intergenic distance and transcription orientation on the level of coexpression of neighboring genes. To further investigate the coexpression of proximate genes, we investigated the level of coexpression of genes in positional clusters identified in the neighbourhood model. Our bioinformatics analyses suggest that a positive correlation exists between the significance of positional gene clusters with the degree of coexpression of genes in the clusters.

## Results

### Proximate genes are coexpressed in the zebrafish genome

**Figure 1:**
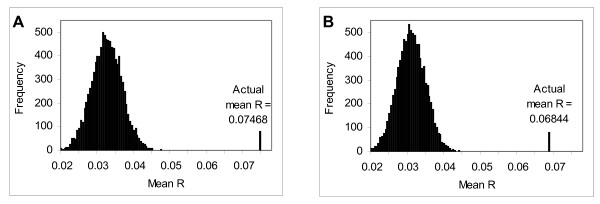
**Distribution of 10,000 mean *R *values calculated from randomized genome**. Each plot shows the distribution of 10,000 mean *R *values. Each mean *R *value is calculated by first randomly permuting the gene order of the genome, and then averaging the *R *values for every pair of neighboring genes in the resulting gene order. The mean R value in the real genome is shown as a single line on each plot. Both plots are based on the same gene expression dataset: (A) the results on the original dataset (average of mean *R *= 0.03086, σ = 0.00384); (B) the results after tandem duplicates are removed (average of mean *R *= 0.03071, σ = 0.00389).

In order to study the coexpression of proximate genes, we analyzed 100 expression datasets derived from Affymetrix microarray experiments. We use the Pearson correlation coefficient (*R*) of two genes to measure the level of their coexpression. The mean *R *of all the neighbouring gene pairs in our dataset is 0.07468 (with standard error 0.00424). This mean value is statistically significant (with *p*-value 0.0001) as it is +11.4 standard deviations from the mean *R *in a randomized genome. In a randomized genome with the same genes and expression values, the mean *R *is only 0.03086 (with standard deviation 0.00384) (Figure 1A). Tandem duplicated genes have identical functions and hence are often highly coexpressed. To eliminate the effects of tandem duplicates on this coexpression study, we removed all members except one in each tandem gene cluster and redid the analysis. After removal of tandem duplicates, the mean *R *became 0.06844 (with standard error 0.00426). It is slightly smaller than the value when all genes are included in the analysis, but still significant (with *p*-value 0.0001, +9.7 standard deviations from the random mean) (Figure 1B).

Surprisingly, the mean *R *is almost identical for zebrafish and *Arabidopsis *genomes [15] when the gene order is randomized, which is about 0.03 with standard deviation 0.004). The underlying cause behind this is unclear. The positive mean value rather than zero could be an effect of the coexpression of the housekeeping genes as suggested in Williams and Bowles [15]. The reason for the identical mean *R *when these two genomes are randomized is probably either that the housekeeping genes show common patterns of expression in different genomes, dominating the mean *R *value, or alternatively, that there is a constant bias or weak autocorrection between all genes in each microarray dataset (see Additional file 1 for detailed discussion and also [11]).

**Figure 2:**
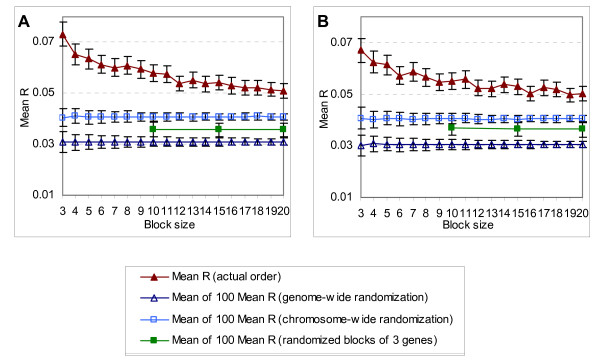
**Mean of pair-wise *R *values in blocks of size 3 to 20 (▲), shown with standard error**. This is compared to the mean of 100 values obtained similarly, each from the same analysis after a random permutation of: (1) the gene order of the entire genome (△); (2) the order of genes in each chromosome (□); (3) the order of non-overlapping blocks of 3 consecutive genes (■). Plots (△), (□) and (■) are shown with standard deviations. The points in (**A**) are from analyses with the full dataset, while (**B**) are from analyses after tandem duplicates are removed.

To further explore the coexpression of proximate genes, we partitioned the genes into non-overlapping blocks of *k *(3 ≤ *k *≤ 20) physically adjacent genes according to their start position. For each gene block, we calculated a mean *R *of the coexpression values; then the mean of all the mean *R*s is calculated and plotted in Figure 2. The degree of coexpression first decreases and then becomes stable when the block size *k *increases from 3 to 20. To verify the significance of the coexpression degree of the genes for each block size, we compared them to what would have been obtained if the genes had been rearranged in each of the following three ways: (1) randomly permuting the gene order over the entire genome; (2) randomly permuting the order of genes within each chromosome; and (3) randomly permuting the order of non-overlapping blocks of 3 consecutive genes. The last rearrangement is used to examine whether the coexpression degree in the larger blocks are dominated mainly by genes that are separated by only one or two genes. The analyses show that there is a significant difference in degree of coexpression between actual and randomized genome (Figure 2). Finally, we remark that the start point for partitioning the genes into non-overlapping blocks has little effect on the analysis presented above because of the way we calculate the mean coexpression value of genes within a block.

### Coexpression of genes within GO classes

Genes are classified into different classes according to the biological processes they are involved in the GO database [22]. We evaluated the mean *R *of the coexpression value of a pair of genes within a GO class that contains 20 or less genes. There are 853 such GO classes. The mean *R *is higher than 0.13, 0.30 and 0.50 in 50%, 25% and 10% of these classes, respectively (see Additional file 2). Thus, genes within a GO class are highly coexpressed as reported in other genomes.

### Distance and coexpression

**Figure 3:**
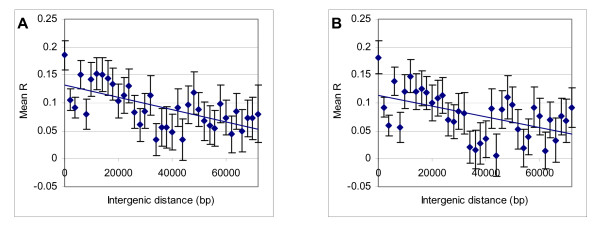
**Mean *R *of gene pairs of up to 50 kbp apart**. Gene pairs of up to 50 kbp apart were binned according to their intergenic distance, shown with regression lines. (A) is from the full dataset, whereas (B) from the resulting dataset after tandem duplicates are removed.

In this analysis, the genes within 50 kilo-base pairs (kbp) from each other are collected, mean *R *values calculated, and the distance between them rounded to the nearest number in 0, 2000, 4000..., 50000. Figure 3 plots the mean of these *R *values against their intergenic distance. There is a clear negative correlation between intergenic distance and degree of coexpression in both the full dataset (regression line *R*^2 ^= 0.50) or after removal of tandem duplicates (regression line *R*^2 ^= 0.46). The correlation is most significant for the gene pairs of distances between 10 kbp to 40 kbp (*R*^2 ^= 0.79, *p *< 0.005 for the full dataset, *R*^2 ^= 0.83, *p *< 0.005 for the dataset without tandem duplicates). Outside of this range, this correlation seems to be absent.

### Gene orientation and coexpression

Genes can be transcripted in two directions, denoted by (→) and (←). Thus, two genes can have: divergent transcription (← →), convergent transcription (→ ←), or parallel transcription (← ← or → →) orientation. We partitioned all the gene pairs into three groups according to transcription orientation. For each orientation class of gene pairs, we calculated the mean *R *value. Regardless of whether tandem duplicates are removed or not, gene pairs with parallel orientation showed the highest degree of coexpression, in both average as well as median values, while gene pairs with convergent orientation showed the lowest degree of coexpression (Table 1). Kruskal-Wallis tests confirmed this effect of orientation (*p *= 0.0267) for the entire dataset, but the effect is insignificant after the removal of tandem duplicates (*p *= 0.2894). This is presumably because most tandem gene pairs have parallel orientation.

**Table 1:** Descriptive statistics for pair-wise comparison of neighboring genes according to orientation of transcription

			**Pearson's correlation coefficient (R)**		**Intergenic distance (bp)**	
	**Orientation**	**N**	**Mean R ± se**	**Median R**	**Mean bp ± se**	**Median bp**

**Analyzed with full dataset**	← →	1681	0.0637 ± 0.0084	0.0238	215221.3 ± 7887.2	88957
	
	→ →/← ←	3418	0.0877 ± 0.0061	0.0547	201669.6 ± 5611.0	75199
	
	→ ←	1678	0.0592 ± 0.0082	0.0238	207196.8 ± 8385.1	75805

**Analyzed w/o tandem duplicates**	← →	1635	0.0618 ± 0.0086	0.0220	219869.4 ± 8382.6	91546
	
	→ →/← ←	3295	0.0762 ± 0.0061	0.0438	209041.5 ± 5714.5	82818
	
	→ ←	1632	0.0594 ± 0.0083	0.0250	216217.3 ± 8729.1	80961

### Positional clustering and the level of coexpression

We have seen from the analyses that a correlation exists between intergenic distance and degree of coexpression. To further the study in this direction, we examined coexpression among the genes in a positional cluster. We adopted the neighbourhood model to identify positional gene clusters and evaluated positional clustering using a method proposed by Li, Lee and Zhang [19]. In the neighbourhood model, two genes are in a cluster if and only if there is a series of genes between them such that the distance between two adjacent genes in the series is less than a specified distance (*D*).

The significance of a positional cluster depends on the value of *D*, the number of genes it contains, and the gene density of its vicinity. In this study, we set *D *to be 25K, which is one-eighth of the average distance between genes (206K for the full dataset, 213K after removal of tandem duplicates. c.f. Table 1). These clusters are described in Additional file 3.

Neighbouring genes within the clusters we identified tend to show a higher degree of coexpression than neighbouring pairs that are not. This observation is consistent with the observation that is mentioned above: a pair of genes within shorter intergenic distance tends to be more coexpressed. Table 2 shows that with only one exception, the mean *R *values for clusters of all sizes are higher than 0.07468, the value of mean *R *of all neighbouring gene pairs in the whole dataset.

**Table 2:** Mean of *R *values for all neighboring gene pairs found within some cluster of size *d *(*d *= 2, 3 ..., 7, > 7).

	**Number of gene pairs**		**Mean R**	
		
**Cluster size (*d*)**	**Analyzed with full dataset**	**Analyzed w/o tandem duplicates**	**Analyzed with full dataset**	**Analyzed w/o tandem duplicates**
2	877	867	0.1051 ± 0.0122	0.0930 ± 0.0121
		
3	560	520	0.0952 ± 0.0152	0.0799 ± 0.0157
		
4	315	282	0.1237 ± 0.0202	0.1148 ± 0.0207
		
5	140	148	0.0739 ± 0.0303	0.0870 ± 0.0306
		
6	125	80	0.1302 ± 0.0328	0.1330 ± 0.0385
		
7	60	48	0.2360 ± 0.0545	0.2505 ± 0.0585
		
> 7	88	62	0.3427 ± 0.0390	0.2456 ± 0.0492

Among the identified positional clusters, there are ten highly significant clusters containing eight or more genes, listed in Table 3. They include hox gene clusters *hoxba *on *Chr3 *and hoxca on *Chr23 *[23], olfactory receptor gene cluster *or1 *on *Chr15*, *cytochrome P450 aromatase *gene cluster *cyp2j *on *Chr20*, and major histocompatibility complex (*Mhc*) gene cluster on *Chr19 *[24,25]. The other five clusters contain a mix of genes from different GO classes. Although these genes have not yet been investigated, they are likely structurally and functionally unrelated. In each of the gene clusters except for three eight-gene clusters on *Chr4*, *Chr19 *and *Chr20*, genes have high level of coexpression.

**Table 3:** The positional clusters which contain at least 8 genes. The *xxx *stands for genes with unknown functions.

***Chr***	**size**	**span**	***p*-value**	**mean *R***	**genes listed in order**
3	10	100K	1.24e-6	0.289	*hoxb1a, hoxb2a, hoxb3a, hoxb4a, hoxb5a, hoxb6a, hoxb7a, hoxb8a, hoxb9a, hoxb10a*

4	8	197K	9.19e-7	0.031	*zgc:86611, psmc2, psmc2, smo, si:dkey-180p18.2, si:dkey-180p18.2, ube2h, nrf1*

5	8	89k	1.36e-5	0.213	*ptges, usp20, zgc:103692, surf5, rpl7a, surf1, surf6l, ccbl1*

13	8	139K	6.39e-5	0.148	*xxx, golga5, rtf1, zgc:113197, zgc:113197, tmem39b, xxx, zgc:123267*

15	17	174K	2.69e-7	0.371	*xxx, or7.1, or2.6, or2.4, xxx, or2.7, xxx, or2.5, or2.1, or2.10, or2.8, xxx, or13.4, or5.1, or5.3, or5.4, or5.2*

19	13	189K	4.12e-7	0.229	*Kifc1, zbtb22, daxx, tpsn, xxx, xxx, xxx, psmb10, psmb11, psmb9a, xxx, brd2, fabgl*

	8	159K	3.04e-5	-0.026	*stk3, zgc:92739, rpl30, laptm4b, lyricl, rrm2b, azin1, atp6v1c1l*

20	8	168K	1.77e-6	-0.075	*zgc:55404, si:dkeyp-55f12.3, itpk1, chga, si:dkey-177p2.3, ahsa1, si:dkey-177p2.6, zgc:92217*

	8	110K	2.61e-4	0.259	*xxx, xxx, paics, cyp2j21, cyp2j22, cyp2j25, cyp2j26, cyp2j28*

23	10	96K	2.12e-5	0.606	*hoxc13a, xxx, hoxc11a, hoxc10a, hoxc9a, hoxc8a, hoxc6a, hoxc5a, hoxc4a, hoxc3a*

It is proposed that evolutionary selection organizes genes according to their biological function so that their expression can be co-ordinately regulated. To test this hypothesis, we use the GO database [22] as a source of annotations of biological processes. The number of clusters formed by genes in some GO class is listed in Table 4 (see Additional file 4 for details). The number is very much reduced when compared to the total number of clusters for each size. Thus, most positional gene clusters we observe are likely not composed of genes with similar biological functions. As suggested by Spellman and Rubin [10], the above hypothesis is not supported.

**Table 4:** Number of positional gene clusters found with intergenic distance *D *= *25K*.

		**Size of gene clusters**						
		
		**2**	**3**	**4**	**5**	**6**	**7**	**> = 8**
**Complete data**	**Number of clusters formed by genes in some GO class**	328	48	12	3	2	0	3
	
	**Total number of clusters**	847	280	105	35	25	10	10

**Data without tandem duplicates**	**Number of clusters formed by genes in some GO class**	322	33	9	1	2	1	1
	
	**Total number of clusters**	867	260	94	37	16	8	8

**Figure 4:**
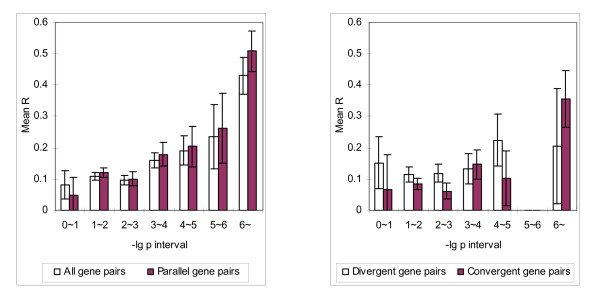
**Mean *R *of neighboring gene pairs in different -lg *p*-value intervals**. Mean *R *values of neighboring gene pairs in -lg *p *intervals. All *p*-values were calculated with *D *= *25K*. Gene pairs grouped into parallel, divergent, and convergent orientations are plotted similarly. There is only one gene pair has -lg *p*-value in the interval 5~6, for both the divergent and convergent cases. They are hence omitted from the plot.

**Figure 5:**
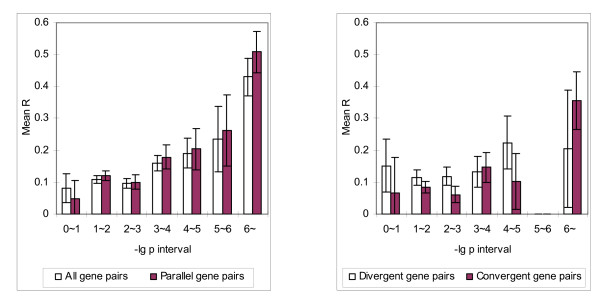
**Results from the same analysis as in Figure **4** after tandem duplicates are removed**.

**Figure 6:**
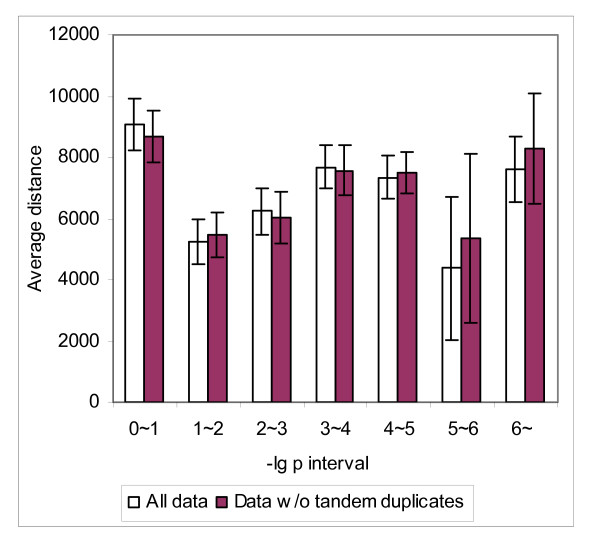
**Average distance between neighboring gene pairs in different -lg *p*-value intervals**.

Finally, we investigated whether there is a correlation between the mean *R *of gene pairs and *p*-value for a positional cluster. With *D *= 25K, we considered all the pairs of the neighbouring genes in the same cluster. We divided the gene pairs into seven categories according to the *p*-value of the clusters to which the gene pairs belong. These seven categories correspond one-to-one to the following intervals of *p*-values: 0~10^-6^, 10^-6^~10^-5^, 10^-5^~10^-4^, 10^-4^~10^-3^, 10^-3^~10^-2^, 10^-2^~10^-1^, 10^-1^~1. To simplify presentation we consider (base 10 logarithm) -lg *p*-value instead of *p*-value, and use the intervals: 0~1, 1~2, 2~3, 3~4, 4~5, 5~6, > 6. We calculated the mean *R* of the neighbouring gene pairs in each category and observed a significant correlation between -lg *p*-value and the degree of coexpression of neighbouring gene pairs, either using the complete dataset (Figure 4) or the dataset after tandem duplicates are removed (Figure 5). This correlation is extremely significant for gene pairs that are transcripted in the parallel orientation. The mean *R* value is as high as 0.5088 (with standard error 0.0642) for the complete dataset and 0.3228 (with standard error 0.1330) even after tandem duplicates are removed. We also observed that at low *p*-value (high -lg *p*-value), more gene pairs in the identified clusters are transcribed in the parallel orientation, even with tandem duplicates (Table 5). We examined a correlation between -lg *p*-value and neighbouring gene distance to find if such a high correlation can be explained with intergenic distance. No such correlation was found (Figure 6).

**Table 5:** Number of neighboring gene pairs found in clusters in different -lg *p*-value intervals (*D *= *25K*).

	**Transcription orientation**	**Number of gene pairs within -lg *p*-value range**						
		
		**0~1**	**1~2**	**2~3**	**3~4**	**4~5**	**5~6**	**6~**
**Analyzed with full dataset**	**All orientations**	64	1101	660	210	79	16	35
	
	**Parallel**	30	541	331	107	48	14	23
	
	**Divergent**	18	237	167	51	17	1	6
	
	**Convergent**	16	323	162	52	14	1	6

**Analyzed w/o tandem duplicates**	**All orientations**	63	1073	586	169	83	14	19
	
	**Parallel**	27	518	291	89	49	12	9
	
	**Divergent**	17	244	152	41	18	1	5
	
	**Convergent**	19	311	143	39	16	1	5

## Discussion

As the zebrafish genome is almost completely sequenced, more and more information has been available for genome-wide analysis. Using public microarray datasets and gene annotation, we investigated, for the first time, the global gene expression patterns in the zebrafish genome. Our results have several implications.

First, proximate genes in the zebrafish genome tend to coexpress at a significant level. This is partially due to tandem genes, which often have high degree of coexpression. The coexpression level decreases with these tandem genes excluded, but still remain significant. These observations are in general comparable – in both effects and magnitude – to the other studies surveyed in [1] and [2]. We measured the degree of coexpression not only between two neighbouring genes, but also among the genes in blocks of sizes from 3 to 20. In each case, the degree of coexpression is significant compared with that of a random genome in which the genes are randomly rearranged.

As shown in other genomes [14,15,26], the degree of coexpression is to some extend influenced by the intergenic distance, and there is evidence of clusters that span 20 coexpressing genes. To investigate this fact further, we examined whether genes in positional clusters of arbitrary span have more significant level of coexpression or not. The average intergenic distance is about 200 kb with or without tandem genes. Using the statistical method proposed by Li, Lee and Zhang [19], we examined the positional gene clusters within which the intergenic distance is less than 25 kb. As shown in Table 3, the genes in these positional clusters usually have higher degrees of coexpression although most of these clusters are composed of genes in different GO classes. We observe ten large positional clusters each having eight or more genes. One of these statistically significant clusters contains 13 highly co-expressed genes in the *Mhc *class I region. Interestingly, Murray et al. [24] noted that the *psmb *genes on the zebrafish *Mhc *class I region concurs with Hughes' hypothesis of a selective advantage to the clustering of genes with similar expression patterns [27]. Moreover, five clusters listed in Table 3 have not been investigated yet. The genes in these newly identified clusters are probably worthy to be investigated biologically.

We also investigated the effect of transcript orientation on the level of coexpression. In yeast [14], human [26], *Arabidopsis *[15], and *C. elegans *[6], it is observed that transcript-divergent neighbouring genes have higher coexpression level than transcript-parallel or transcript-convergent neighbouring genes. Our study finds that transcript-parallel or transcript-divergent neighbouring genes have higher coexpression level than transcript-convergent genes in the zebrafish genome. The fact that the transcript-convergent neighbouring genes have the lowest level of coexpression is consistent with the studies that are just mentioned. This fact is likely related to 5' *cis*-regulatory elements [6]. Only transcript-parallel or -divergent gene pairs can be driven by a 5' cis-regulatory element. For example, the genes in the identified positional cluster in the *Mhc *class I region on Ch19 are believed to be coregulated from shared bidirectional promoters [24]. However, that the transcript-parallel neighbouring genes have a higher level of coexpression than the transcript-divergent neighbouring genes could be special to the zebrafish genome as such a fact has not been reported in other genomes to our best knowledge. The underlying cause for it could be that there are less bidirectional promoters in the zebrafish genome than in mammalian genomes; it may also be due to the fact that the tandem neighbouring genes that have parallel orientation are strongly coexpressed in the zebrafish genome, which may result from our analysis being done on a partial list of genes and incomplete positional information. When the zebrafish genome is completely sequenced in the near future, repeating our analysis will definitely give a better picture of the influence of transcript orientation on the coexpression level of zebrafish genes.

## Conclusion

In summary, we have observed that gene order of the zebrafish is non-random. In addition, the statistical significance of genes' positional clustering is positively correlated to coexpression degree. These facts suggest that the clustering of genes may be subjected to selection forces that favour having coexpressing genes in close proximity.

## Methods

### Microarray data sources

Our gene-expression datasets are compiled from several previous studies with the Affymetrix GeneChip^® ^Zebrafish Genome Array (GeneChip 430), which contains 39,000 *Danio rerio *transcripts. These microarray data were used to study the transcriptional changes of genes in embryonic development and divided into the following four groups:

(i) Nine expression datasets based on experiments on zebrafish embryonic fibroblast cell lines ZF4 and PAC2 (with accession id E-MEXP-736 in ArrayExpress) [28]. They are the gene expression profiles of these two cell lines in cultures with and without the presence of serum.

(ii) Two expression datasets (id E-MEXP-737) derived from experiments on the 24-hour embryos from the Tuebingen cell line [28].

(iii) Forty-two expression datasets (id E-MEXP-758) from analysis of the transcriptional response to TCDD at different stages [29]. They were used to identify the gene expression changes in the heart and other tissues of zebrafish larvae at 1 h, 2 h, 4 h and 12 h after exposure to TCDD beginning at 72 h fertilization, and

(iv) Forty-one expression datasets collected at the Lab of Functional Genomics, the Institute of Molecular and Cell Biology, Singapore [30-32]. The microarray data in [30] were collected in the experiments with RNA extracted from wild type AB and *def*^*hi*429 ^mutant embryos after 5-day fertilization. In [31], microarray gene expression profiles of the liver and the remaining liver-free body of adult zebrafish (wild type AB strain) were used to study the regulation mechanism of liver-enriched genes. In [32], microarray data were obtained from the experiments with RNA samples extracted from five embryos for gene expression profiling of the 18-somite zebrafish cloche mutant, in which development of hematopoietic lineage is severely impaired.

Microarray experiment database ArrayExpress is available at EMBL-EBI [33]. These available datasets were preprocessed by using the invariant set normalization method [34].

### Locating expressed genes in Zebrafish genome

Expressed genes were identified using the Ensembl database. The clone sequences in Affymetrix Zebrafish Genome Array were aligned back to the zebrafish genomic sequence (available from [35]) using BLAST program. This results in 6802 expressed zebrafish genes. The positional information of these genes was then extracted to arrange the genes for analysis.

### Identification of biological processes genes are involved in

The zebrafish GO terms are obtained along with the genomic sequences from the Ensembl database [35]. It annotates the 6802 genes into 1722 GO classes. The correspondence between gene IDs and Affymetrix Zebrafish DB IDs is obtained similarly.

### Removal of tandem genes

We used the same criterion to remove tandem duplicates as in [3]. Two genes are considered as tandem duplicate if they are within 100 genes from each other and aligned by BLAST with E-value less than 0.2. After a tandem gene cluster was detected, we removed all but one gene from the analysis. After removal of 215 tandem genes, 6587 genes remained for further analysis.

### Measuring the level of coexpression

Pearson's correlation coefficient (*R*) is used to measure the level of coexpression between two genes. For two genes *X *and *Y *with expression values (*x*_1_, *x*_2_,... *x*_*n*_) and (*y*_1_, *y*_2_,..., *y*_*n*_) in *n *microarray experiments respectively, *R*(*X*, *Y*) is computed as

$$\frac{\sum_{i}\left(x_{i}-\bar{x})(y_{i}-\bar{y}\right)}{\sqrt{\sum_{i}{\left(x_{i}-\bar{x}\right)}^{2}\sum_{i}{\left(y_{i}-\bar{y}\right)}^{2}}}$$

where $\bar{x}$ and $\bar{y}$ are the mean expression value of *X *and *Y *in an experiment respectively.

The significance of mean *R *calculated from the real data was estimated by comparing it with the mean *R*s for 10000 random genomes. In each random genome, genes were rearranged though a series of transposes.

To analyze the level of coexpression among the multiple proximate genes, we divided the genes in the Zebrafish genome into non-overlapping blocks, each of *k *consecutive genes, where *k *is a fixed integer from 3 to 20. For a block of *k *genes, there are *k*(*k *- 1)/2 pairs of genes; the mean *R *of these pairs is used to measure the degree of coexpression among the genes in the block, called the block R. The mean block *R *was compared with mean block *R*s calculated from the randomized genome.

### Analysis of positional gene clusters

We examined positional gene clusters in the neighbourhood model. In the neighbourhood model, two genes x and y are in a cluster if and only if the distance between any two adjacent genes locating between x and y is less than a fixed threshold (*D*). For a cluster of *n *genes, its *p*-value is equal to (1 - *e*^-*αD*^)^*n*^, where *α *is the gene density in a considered region [19]. Since gene density varies in the zebrafish genome, we set α to be the gene density in the 2 Mbp region around the gene cluster.

This formula is derived under the assumption that the (start) position of a gene is uniformly distributed. This assumption is obviously invalid in a whole chromosome since there are gene dense and sparse regions in a genome. Thus, we focus on the 2 Mbp region centred on the gene cluster to be analyzed.

## Abbreviations

SAGE: Serial Analysis of Gene Expression; GO: Gene Ontology; TCDD: tetrachlorodibenzo-*p*-dioxin.

## Authors' contributions

WW prepared the microarray data and performed initial analyses. YKN performed the analyses herein. LXZ conceived of the study, coordinated the analysis. YKN and LXZ prepared the final manuscript. All authors read and approved the final manuscript.

## Supplementary Material

Additional file 1 **Analysis of non-zero positive mean *R* value in randomized genome and other discussions.** It contains the analysis of mean *R* in randomized genome and other studies relevant to, but not written in this manuscript. Click here for file

Additional file 2 **Coexpression analysis of genes with GO classes.** It contains spreadsheet data from analysis of coexpression of genes within GO classes. Click here for file

Additional file 3 **Positional gene clusters together with their *p*-values and co expression mean *R*.** It contains the analysis of positional gene clusters for *D* = 25K with/without tandem duplicated genes. Click here for file

Additional file 4 **Positional clusters formed by genes with GO classes.** It contains spreadsheet data from analysis of positional clusters that are composed of genes within GO classes. Click here for file
