# Supplementary material for: Positive correlation between gene coexpression and positional clustering in the zebrafish genome
Source: BMC Genomics. 2009 Jan 22;10:42. doi: 10.1186/1471-2164-10-42 (PMC2654907; doi:10.1186/1471-2164-10-42)
Supplement: Additional file 1 — Analysis of non-zero positive mean R value in randomized genome and other discussions. It contains the analysis of mean R in randomized genome and other studies relevant to, but not written in this manuscript. [file 1471-2164-10-42-S1.pdf]

Proximate genes are coexpressed in the Zebrafish genome

Non-zero positive mean R value in randomized genome

Studying expression data from *Arabidopsis* Affymetrix microarrays, Williams and Bowles (2004) reported a mean R of ~0.03 (standard deviation ~0.004) for neighboring gene pairs when the gene order is randomized. We obtained very similar values for the *Danio rerio* genome using Affymetrix microarrays. The fact that this value is positive instead of zero is possibly due to the influence of the *housekeeping genes* which are known to coexpress. Alternatively, if the arrays had been influenced by biases that are constant to each array (but different for each different array), such that the expressions in each array is changed by the same value, then every pair of genes will tend to show more correlation, and hence resulting in a greater than zero average of mean R.

To test this hypothesis, we first note that such a bias will have larger effects on the coexpression of genes with expression levels that fluctuate less**.** Hence, we can expect the mean R to be closer to zero if the test is repeated with these relatively *stable* genes removed.

We identify three kinds of genes that by the above considerations should theoretically correlate better than typical genes. Furthermore, if their presence is what contributed to the higher than zero mean R, then after their removal, analyses using the remaining genes should result in lower degrees of coexpression. For each array X we denote the maximum expression of the array by X_{max}; the minimum expression of the array X_{min}; and the expression of the array for gene *g* by X_g.

1. A gene *g* is *expressed above level N* iff for every array X, $(X_g - X_{min}) / (X_{max} - X_{min}) \geq N$
2. A gene *g* is *expressed below level N* iff for every array X, $(X_g - X_{min}) / (X_{max} - X_{min}) \leq N$
3. A gene is *stable below level N* iff for any two arrays X, X’,

$$\left| ((X_g - X_{min}) / (X_{max} - X_{min})) - ((X'_g - X'_{min}) / (X'_{max} - X'_{min})) \right| \leq N.$$

The first criterion attempts to identify, to very limited extend, the housekeeping genes. Criteria 2 and 3 define genes that are relatively stable. In Table 1, we show the number (and the corresponding percentage) of genes that fit these criteria for different values of *N*. Only ~9% of the genes can be considered to be of the 1st kind even at level 0.01.

For each set of the genes that fulfill each of the criteria at a different level we obtained 10000 mean R values, each with a different randomization of the gene order. As expected, the results show significant degree of coexpression among the genes of each of the three different kinds, with the 1st kind of genes (housekeeping genes) showing the most significant degree of coexpression (Table 2). Furthermore, mean R values become closer to zero if the 2nd or 3rd kinds

of genes are removed (Table 3), showing that the inclusion of these genes contributed to the higher than zero tendency of the mean R.

However, when the genes of the 1st kind are removed, the mean R values increased in average. This suggests that the genes of the 1st kind may have expression patterns that are different from the rest of the genes.

We note an implication of this result. If the mean R values obtained after randomizing the gene order are increased due to biases in the microarray data, then these values would be closer to zero for a set of microarrays that are relatively unbiased. In which case, larger values of the mean R could be used as an indicator for the existence of constant biases in the microarray data. On the other hand, mean R values closer to zero could be used to support the claim that there is not too much bias in the data.

Table 1 Percentage of genes at different levels of each criterion.

		Percentage of genes	
	N	**Analyzed with full data set**	**Analyzed w/o tandem duplicates**
**Expressed above level *N***	**0.005**	**19.5**	**19.8**
	**0.01**	**8.8**	**8.9**
	**0.02**	**4.2**	**4.3**
	**0.04**	**2.2**	**2.3**
	**0.08**	**1.3**	**1.3**
	**0.16**	**0.8**	**0.8**
**Expressed below level *N***	**0.01**	**22.9**	**22.2**
	**0.02**	**41.7**	**40.5**
	**0.08**	**80.2**	**80.2**
	**0.16**	**90.1**	**90.2**
	**0.24**	**93.8**	**93.9**
	**0.32**	**95.4**	**95.7**
**Stable below level *N***	**5**	**71.5**	**71.4**
	**10**	**85.0**	**85.3**
	**20**	**92.6**	**92.8**
	**30**	**95.4**	**95.7**

Table 2 For each of the criteria and each of the selected levels (*N*), the table shows the mean of 10000 mean R values (shown with standard deviations). Each mean R value is the average of the R values of all neighboring gene pairs, computed after removing the genes that do not fulfill the criteria from the genome and then having the gene order randomly permuted.

	N	Mean of mean R	
		Analyzed with full data set	Analyzed w/o tandem duplicates
Genes expressed above level *N*	0.005	0.08640 ± 0.00915	0.08553 ± 0.00936
	0.01	0.09713 ± 0.01423	0.09674 ± 0.01430
	0.02	0.14547 ± 0.01979	0.14700 ± 0.02036
	0.04	0.23922 ± 0.02287	0.24883 ± 0.02309
Genes expressed below level *N*	0.08	0.04034 ± 0.00416	0.04016 ± 0.00423
	0.16	0.03737 ± 0.00397	0.03693 ± 0.00399
	0.24	0.03534 ± 0.00397	0.03513 ± 0.00396
	0.32	0.03480 ± 0.00386	0.03455 ± 0.00395
Genes stable below level *N*	5	0.04749 ± 0.00429	0.04660 ± 0.00438
	10	0.03904 ± 0.00409	0.03884 ± 0.00409
	20	0.03595 ± 0.00388	0.03569 ± 0.00392
	30	0.03492 ± 0.00389	0.03458 ± 0.00394

Table 3 For each of the criteria and each of the selected levels (*N*), the table shows the mean of 10000 mean R values (shown with standard deviations). Each mean R value is the average of the R values of all neighboring gene pairs, computed after removing the genes that fulfill the criteria from the genome and then having the gene order randomly permuted.

	N	Mean of mean R	
		Analyzed with full data set	Analyzed w/o tandem duplicates
Genes expressed above level *N*	0.005	0.03653 ± 0.00422	0.03646 ± 0.00429
	0.01	0.03554 ± 0.00393	0.03552 ± 0.00403
	0.02	0.03448 ± 0.00383	0.03446 ± 0.00397
	0.04	0.03370 ± 0.00378	0.03356 ± 0.00393
Genes expressed below level *N*	0.08	0.01561 ± 0.00947	0.01628 ± 0.00961
	0.16	0.01062 ± 0.01358	0.01287 ± 0.01405
	0.24	0.00994 ± 0.01771	0.01194 ± 0.01823
	0.32	0.01609 ± 0.02054	0.02293 ± 0.02191
Genes stable below level *N*	5	0.01711 ± 0.00777	0.01760 ± 0.00798
	10	0.01016 ± 0.01117	0.01109 ± 0.01127
	20	0.00640 ± 0.01611	0.00948 ± 0.01667
	30	0.01567 ± 0.02103	0.02145 ± 0.02212

Positional clustering and degree of coexpression

Information on Clusters Found with Neighborhood Model

Table 1 Numbers of clusters of sizes 2 to >7 identified on each chromosome (from 1 to 25) at D=25K.

Cluster size	Number of clusters on chromosome																								
	1	2	3	4	5	6	7	8	9	10	11	12	13	14	15	16	17	18	19	20	21	22	23	24	25
2	38	55	35	28	51	40	60	40	30	30	30	30	38	42	19	37	33	23	27	50	28	26	34	29	24
3	15	10	17	6	21	11	15	15	11	10	7	11	11	11	8	10	11	7	17	12	13	8	7	9	7
4	4	6	9	5	7	5	2	5	4	3	6	5	5	3	3	4	1	4	3	7	5	6	2	0	1
5	0	0	4	3	3	3	2	1	1	0	1	2	1	0	1	4	1	1	2	2	0	1	0	1	1
6	1	2	3	1	2	1	2	0	1	1	1	0	2	1	0	1	1	0	2	1	1	0	1	0	0
7	1	2	0	0	1	0	1	0	0	0	0	0	0	1	0	0	1	0	1	1	0	0	0	0	1
>7	0	0	1	1	1	0	0	0	0	0	0	0	1	0	1	0	0	0	2	2	0	0	1	0	0

Table 2 Numbers of clusters of sizes 2 to >7 found on each chromosome (from 1 to 25) at D=25K (analyzed without tandem duplicates).

Cluster size	Number of clusters on chromosome																								
	1	2	3	4	5	6	7	8	9	10	11	12	13	14	15	16	17	18	19	20	21	22	23	24	25
2	38	52	37	28	52	41	56	38	30	30	27	26	38	40	22	36	35	23	29	51	28	26	32	26	26
3	14	11	13	6	18	10	15	13	8	10	8	12	11	11	8	12	9	8	14	13	10	7	6	9	4
4	3	6	8	5	6	5	2	5	4	4	5	4	4	3	1	3	1	2	3	6	5	5	3	0	1
5	1	0	5	3	2	2	2	1	1	0	1	2	2	1	1	4	1	1	1	3	0	1	0	1	1
6	1	1	2	1	2	1	2	0	0	0	1	0	0	0	0	0	1	0	2	1	1	0	0	0	0
7	0	2	0	0	1	0	1	0	0	0	0	0	0	1	0	0	1	0	1	0	0	0	0	0	1
>7	0	0	1	1	1	0	0	0	0	0	0	0	1	0	0	0	0	0	2	1	0	0	1	0	0

Table 3 Average cluster lengths for clusters of different sizes (shown with standard deviations).

Cluster size (*d*)	Average cluster length (bp)	
	Analyzed with full data set	Analyzed w/o tandem duplicates
4	85865 ± 48396	104404 ± 49786
5	93713 ± 38961	141621 ± 70091
6	130101 ± 74066	143963 ± 59018
7	118760 ± 44892	178773 ± 70713
>7	142367 ± 40620	207858 ± 110245
